# Understanding gingival pigmentation; insights from the Malaysian population: A cross-sectional study

**DOI:** 10.6026/973206300222848

**Published:** 2026-05-31

**Authors:** Tina Varghese, Prebha Manickam, Abin Varghese, Muhammad Khairan AI-Hilal Bin Muhammad Reezal, Nuraminah Binti Mohd Fadhil, Sharina Aqila Binti Shaharzat

**Affiliations:** 1 Department of Periodontology, Penang International Dental College, Butterworth, Penang, Malaysia; 2 Department of Oral Surgery, Penang International Dental College, Butterworth, Penang, Malaysia; 3 Department of Dentistry, Shah Alam, Selangor, Ministry of Health, Malaysia; 4 Department of Dentistry, Subang Jaya, Ministry of Health, Malaysia; 5 Department of Dentistry, Merlimau, Melaka, Ministry of Health, Malaysia

**Keywords:** Ethnicity, gingival pigmentation, Malaysia, smoking, variability

## Abstract

Gingival pigmentation varies across populations, yet limited research exists on its prevalence and contributing factors within the Malaysian population. Therefore, it is of interest to investigate the prevalence, distribution and factors influencing gingival pigmentation among 100 participants from different ethnic groups in Malaysia. Data were collected through clinical examinations and questionnaires assessing demographic, lifestyle and ethnic variables. The study found significant ethnic variations, with Malays and Indian participants exhibiting higher pigmentation levels, particularly among smokers. Thus, we document comprehensive insights into gingival pigmentation patterns in Malaysia, which may inform clinical practice and future research.

## Background:

Gingival pigmentation, also known as gum pigmentation, refers to the color variation observed in the gingival tissue, particularly in terms of darkened areas or spots, which can range from light brown to deep blue-black [1]. While some degree of pigmentation is natural and physiological, variations in the extent, depth and type of pigmentation can occur due to several factors, including genetic influences, ethnic backgrounds and environmental exposures [2]. The phenomenon has piqued the interest of researchers due to its implications not only in cosmetic dentistry but also in the broader field of oral health. Gingival pigmentation can vary greatly among different populations, reflecting distinct patterns related to race, ethnicity and genetic predisposition [3, 4]. In the context of the Malaysian population, gingival pigmentation has not been extensively studied, especially with respect to its distribution, underlying causes and impact on individuals' perceptions of their oral health. Malaysia is a multi-ethnic country, comprising a wide range of ethnic groups, including Malays, Chinese, Indians and indigenous populations [5]. The scientific community has long recognized that the presence of melanin, a natural pigment found in the skin and mucous membranes, plays a critical role in gingival pigmentation. In individuals with darker skin tones, melanin production is often higher, resulting in darker gingival tissue [6]. Conversely, individuals with lighter skin tones tend to have lighter gingiva. However, the exact mechanisms governing this pigmentation process are not fully understood and remain a subject of ongoing research. Factors such as age, gender, oral hygiene practices and exposure to environmental elements like tobacco and medications also appear to influence gingival color [7]. However, the extent to which these factors contribute to variations in pigmentation in the Malaysian population remains unclear. Recent studies conducted in various parts of the world have shown that gingival pigmentation can be influenced by a range of factors such as ethnicity, with darker pigmentation more commonly observed among individuals of African, Asian and Middle Eastern descent [8]. While some studies have focused on particular ethnic groups within Malaysia, more comprehensive research on the national scale is needed to better understand the diverse factors at play. Such understanding is crucial for dental practitioners who may encounter a variety of pigmentation patterns in their patients. Additionally, an improved understanding of gingival pigmentation can enhance patient care and treatment outcomes by allowing for better diagnosis, appropriate treatment planning and the management of aesthetic concerns related to the gingiva [9]. Therefore, it is of interest to evaluate the prevalence, distribution and contributing factors of gingival pigmentation in the Malaysian population, as it will provide valuable insights for clinical practice and future research in this area.

## Methodology:

The methodology of this study involved a cross-sectional design to evaluate the prevalence, distribution and contributing factors of gingival pigmentation among 100 participants in Malaysia. The study sample was selected using a stratified random sampling technique to ensure representation from various ethnic groups, including Malays, Chinese, Indians and indigenous populations. Participants were recruited from local dental clinics and community health centers across different regions of Malaysia. Inclusion criteria included individuals aged between 18 and 60 years with no history of systemic diseases, periodontal diseases or previous gingival surgeries that could interfere with the pigmentation of the gingiva. Individuals who had undergone treatments such as gingival bleaching or use of melanin -reducing agents were excluded from the study to avoid skewing the results. Upon obtaining written informed consent from each participant, data were collected through clinical examinations and self-administered questionnaires. The clinical examination was performed by a trained dentist to assess the presence and extent of gingival pigmentation. The pigmentation was classified based on color, location and distribution, using a standardized scale that ranges from mild pigmentation (light brown) to dark pigmentation (blue-black). Gingival pigmentation was recorded using intraoral photographs to accurately document the visual appearance and severity of pigmentation in the anterior and posterior regions of the maxilla and mandible. Alongside the clinical examination, participants were asked to complete a questionnaire designed to gather demographic information, including age, gender, ethnic background and smoking habits. The questionnaire also inquired about the participants' oral hygiene practices, use of medications and any history of environmental exposures that might influence gingival pigmentation, such as tobacco use or frequent consumption of certain foods or beverages. The data from the questionnaires were used to identify potential risk factors that could contribute to variations in gingival pigmentation. Data analysis was performed using statistical software to determine the prevalence of gingival pigmentation in the sample and explore the relationship between pigmentation and various demographic, genetic and environmental factors. Descriptive statistics were used to summarize the data, while chi-square tests were conducted to assess the association between gingival pigmentation and categorical variables such as ethnicity, age, gender and smoking status. A multivariate analysis was performed to control for potential confounders and better understand the factors contributing to pigmentation variations. This study adhered to ethical guidelines, ensuring confidentiality and privacy for all participants. Ethical approval was obtained from the relevant institutional review board before the commencement of the study. The results of this study are expected to provide a comprehensive understanding of gingival pigmentation in the Malaysian population, highlighting the role of ethnicity, lifestyle factors and other contributing variables.

## Results:

The prevalence of gingival pigmentation varies across different ethnic groups. The Malay ethnicity has the highest percentage of individuals with mild pigmentation (50%) and moderate pigmentation (26.67%), while the Chinese ethnicity shows a higher percentage of individuals with no pigmentation (15 out of 30). Indians have a moderate distribution, with 12 individuals (48%) exhibiting mild pigmentation and 28% with moderate pigmentation. The Indigenous group shows a relatively balanced distribution of mild (33.33%), moderate (33.33%), and severe pigmentation (20%) (Table 1). In terms of severity, Malay individuals display the highest percentage of mild pigmentation (50%), followed by moderate pigmentation (26.67%) and severe pigmentation (6.67%). Chinese individuals exhibit a more even distribution, with 26.67% showing mild pigmentation and 16.67% showing moderate pigmentation. The Indian g roup also shows a higher percentage of mild pigmentation (48%) and moderate pigmentation (28%). In contrast, the Indigenous group shows an equal distribution between mild and moderate pigmentation (33.33%) and a higher percentage of severe pigmentation (20%) (Table 2). The prevalence of gingival pigmentation severity varies significantly with age. Among individuals younger than 30 years, 45% exhibit mild pigmentation, 25% exhibit moderate pigmentation, and 5% have severe pigmentation. In the 30-40 year age group, the percentage of moderate pigmentation increases to 30%, with 35% having mild pigmentation and 15% shows severe pigmentation. The older age group (>40 years) shows a high prevalence of moderate pigmentation (45%) and severe pigmentation (20%), with a lower percentage of mild pigmentation (20%) (Table 3). In smokers, the distribution of gingival pigmentation shows a higher percentage of moderate pigmentation (18%) and mild pigmentation (10%) compared to non-smokers. Smokers also show a higher prevalence of severe pigmentation (6%). Non-smokers, however, exhibit a higher percentage of mild pigmentation (30%) and a lower incidence of moderate pigmentation (7%), with only 4% exhibiting severe pigmentation (Table 4).

The results of this study confirm that gingival pigmentation is a common occurrence in the Malaysian population, with a prevalence rate of 75%. The pigmentation is notably more pronounced in the Malay, Indian and Indigenous ethnic groups, which aligns with existing literature that reports darker pigmentation in populations with higher melanin production. The ethnic differences in pigmentation severity highlight the influence of genetic factors, with Malay and Indian participants exhibiting higher levels of moderate to severe pigmentation compared to Chinese and Indigenous participants. Age also appears to be a contributing factor, with older individuals showing more significant pigmentation. This may be related to cumulative environmental exposure or age-related changes in melanin production. The gender differences, although not statistically significant, suggest a potential trend for higher pigmentation in males, which could be explored further in future studies. Smoking emerged as a strong predictor of gingival pigmentation, with smokers showing a significantly higher prevalence of moderate to severe pigmentation. This finding aligns with studies that link smoking to increased melanin deposition in the gingiva, likely due to the irritative effects of tobacco on the oral mucosa. Overall, these findings provide important insights into the factors influencing gingival pigmentation in Malaysia, emphasizing the role of ethnicity, age, gender and smoking. Understanding these factors is crucial for improving clinical practice and guiding aesthetic dental treatments.

## Discussion:

The findings of the present study on gingival pigmentation in a Malaysian cohort can be compared with several previously published studies, highlighting both similarities and differences in patterns of prevalence and associated factors. In a cross-sectional study by Khayamali *et al.* (2025) [10], gingival pigmentation was examined in a clinical population of 380 individuals, where a significant association was found between pigmentation and age, though not with gender. Similar to our results, Khayamali *et al.* reported higher pigmentation with increasing age, reflecting age related accumulation of melanin and possible chronic environmental exposures such as smoking or dietary influences that intensify pigmentation over time. A study by Verma *et al.* (2022) [11] among pre-school children in India demonstrated a positive correlation between gingival pigmentation and age, sex and skin tone, finding that darker skin tones were associated with more intense pigmentation. This aligns with our observations that ethnicity and inherent pigmentation characteristics significantly influence gingival coloration; however, the Verma study involved young children, while our research focused on adults, suggesting that such correlations persist beyond early life and may even strengthen with age. Mirdad *et al.* (2023) [12] investigated gingival melanin pigmentation in a large sample and identified statistically significant relations between pigmentation intensity, skin colour and pigmentation extent. Consistent with our findings, this study reported that darker skin tones exhibited heavier pigmentation, reinforcing the concept that melanin distribution in gingival tissues parallels cutaneous pigmentation gradients across populations. In contrast, Santa Packyanathan and Lavanya (2019) [13] assessed gingival melanin pigmentation among Indian pediatric subjects and found no significant association of pigmentation distribution or intensity with age or sex, although skin colour did influence intensity. This partial divergence from our results specifically regarding age and gender correlations could be due to differences in age groups studied and developmental variations in melanin activity, emphasizing that pigmentation determinant may vary across the lifespan and populations. Moreover, a broader comparative perspective comes from Masilana *et al.* (2017) [14], who reported on physiological oral melanin pigmentation in a South African multi ethnic sample. They found that prevalence varied substantially across ethnic groups with highest rates among individuals of African descent mirroring our observation that ethnically linked pigmentation differences are significant and suggesting that genetic background plays a major role in pigmentation patterns globally.

## Conclusion:

We show significant ethnic, age and smoking-related variations in gingival pigmentation among the Malaysian population. Ethnicity, particularly in Malay and Indian groups, plays a crucial role in determining pigmentation levels. These findings offer valuable insights for clinicians in diagnosing and managing gingival pigmentation in diverse populations.

## Figures and Tables

**Table 1:** Prevalence of gingival pigmentation by ethnicity

**Ethnicity**	**No Pigmentation (n=25)**	**Mild Pigmentation (n=40)**	**Moderate Pigmentation (n=25)**	**Severe Pigmentation (n=10)**	**Total (n=100)**
Malay	5	15	8	2	30
Chinese	15	8	5	2	30
Indian	3	12	7	3	25
Indigenous	2	5	5	3	15
Total	25	40	25	10	100

**Table 2:** Severity of gingival pigmentation by ethnicity

**Ethnicity**	**Mild Pigmentation (%)**	**Moderate Pigmentation (%)**	**Severe Pigmentation (%)**
Malay	50%	26.67%	6.67%
Chinese	26.67%	16.67%	6.67%
Indian	48%	28%	12%
Indigenous	33.33%	33.33%	20%

**Table 3:** Association of age with gingival pigmentation severity

**Age Group**	**Mild Pigmentation (%)**	**Moderate Pigmentation (%)**	**Severe Pigmentation (%)**
<30 years	45%	25%	5%
30-40 years	35%	30%	15%
>40 years	20%	45%	20%

**Table 4:** Gingival pigmentation severity in smokers and non-smokers

**Smoking Status**	**No Pigmentation (n=25)**	Mild Pigmentation (n=40)	**Moderate Pigmentation (n=25)**	**Severe Pigmentation (n=10)**	**Total (n=100)**
Smokers	6	10	18	6	40
Non-Smokers	19	30	7	4	60
Total	25	40	25	10	100
